# T2-Imaging Changes in the Nigrosome-1 Relate to Clinical Measures of Parkinson’s Disease

**DOI:** 10.3389/fneur.2016.00174

**Published:** 2016-10-20

**Authors:** Katherine A. Fu, Romil Nathan, Ivo D. Dinov, Junning Li, Arthur W. Toga

**Affiliations:** ^1^Laboratory of Neuro Imaging, Stevens Neuroimaging and Informatics Institute, University of Southern California, Los Angeles, CA, USA; ^2^Keck School of Medicine, University of Southern California, Los Angeles, CA, USA; ^3^Statistics Online Computational Resource, Health Behavior and Biological Sciences, University of Michigan, Ann Arbor, MI, USA

**Keywords:** movement disorders, Parkinson’s disease, MRI, nigrosome-1

## Abstract

**Background:**

The nigrosome-1 region of the substantia nigra (SN) undergoes the greatest and earliest dopaminergic neuron loss in Parkinson’s disease (PD). As T2-weighted magnetic resonance imaging (MRI) scans are often collected with routine clinical MRI protocols, this investigation aims to determine whether T2-imaging changes in the nigrosome-1 are related to clinical measures of PD and to assess their potential as a more clinically accessible biomarker for PD.

**Methods:**

Voxel intensity ratios were calculated for T2-weighted MRI scans from 47 subjects from the Parkinson’s Progression Markers Initiative database. Three approaches were used to delineate the SN and nigrosome-1: (1) manual segmentation, (2) automated segmentation, and (3) area voxel-based morphometry. Voxel intensity ratios were calculated from voxel intensity values taken from the nigrosome-1 and two areas of the remaining SN. Linear regression analyses were conducted relating voxel intensity ratios with the Movement Disorder Society-Unified Parkinson’s Disease Rating Scale (MDS-UPDRS) sub-scores for each subject.

**Results:**

For manual segmentation, linear regression tests consistently identified the voxel intensity ratio derived from the dorsolateral SN and nigrosome-1 (IR2) as predictive of nBehav (*p* = 0.0377) and nExp (*p* = 0.03856). For automated segmentation, linear regression tests identified IR2 as predictive of Subscore IA (nBehav) (*p* = 0.01134), Subscore IB (nExp) (*p* = 0.00336), Score II (mExp) (*p* = 0.02125), and Score III (mSign) (*p* = 0.008139). For the voxel-based morphometric approach, univariate simple linear regression analysis identified IR2 as yielding significant results for nBehav (*p* = 0.003102), mExp (*p* = 0.0172), and mSign (*p* = 0.00393).

**Conclusion:**

Neuroimaging biomarkers may be used as a proxy of changes in the nigrosome-1, measured by MDS-UPDRS scores as an indicator of the severity of PD. The voxel intensity ratio derived from the dorsolateral SN and nigrosome-1 was consistently predictive of non-motor complex behaviors in all three analyses and predictive of non-motor experiences of daily living, motor experiences of daily living, and motor signs of PD in two of the three analyses. These results suggest that T2 changes in the nigrosome-1 may relate to certain clinical measures of PD. T2 changes in the nigrosome-1 may be considered when developing a more accessible clinical diagnostic tool for patients with suspected PD.

## Introduction

There has been recent interest in determining a reliable *in vivo* biomarker for Parkinson’s disease (PD), a neurodegenerative disorder characterized by motor and non-motor symptoms. The hallmark symptoms of PD, such as resting tremors, bradykinesia, rigidity, and postural instability, are related to dopamine (DA) deficiency (1, 2). An ideal imaging marker is expected to reflect progressive loss of dopaminergic neurons. For such purpose, the nigrosomes within the substantia nigra (SN) region are of particular interest, because they have the highest density of dopaminergic neurons (3). The largest nigrosome is the nigrosome-1, and it is lens-shaped and situated along the rostral/caudal axis of the SN in its dorsal part, at the caudal and intermediate levels (4, 5).

Recent studies noted that high-resolution 7 and 3 T T2*-susceptibility-weighted (SWI) magnetic resonance imaging (MRI) can directly visualize the nigrosome-1 in healthy controls due to the SWI sensitivity for iron (5, 6). However, T2-weighted imaging is more commonly used in routine MRI protocols than SWI. T2-weighted imaging, being sensitive to local magnetic field inhomogeneities, is also modified in the presence of iron, with previous studies suggesting its potential to serve as a non-invasive estimate of iron content in the brain (7–9). Previous studies have implicated that changes in iron levels in the SN of the PD-affected brain may have an influence on the selective and progressive dopaminergic neurodegeneration seen in PD (10, 11). As progressive dopaminergic neurodegeneration is characteristic of PD, T2-weighted images may be able to detect the change in iron content in the nigrosome-1 of PD subjects. Using T2-weighted images from both PD subjects and healthy controls, we related voxel intensity ratios derived from the nigrosome-1 and two other regions of the SN with clinical measures of PD to determine whether the nigrosome-1 can serve as a more readily accessible, potential biomarker for PD, thereby serving as an indicator of disease progression.

## Materials and Methods

### Dataset and Study Population

Data used in the preparation of this article were obtained from the Parkinson’s Progression Markers Initiative (PPMI) database (www.ppmi-info.org/data). The PPMI is a multi-center trial involving 33 centers in North America, Europe, Israel, and Australia for 3–5 years with a primary objective to identify clinical, imaging, and biologic markers of PD progression for use in clinical trials of disease-modifying therapies. The sample consisted of a total of 47 subjects, with 17 healthy controls and 30 patients with PD. Mean age (±SD) was 61.1 (±10.2) years. There were 21 females and 26 males. Subjects’ disease status was determined according to the PPMI selection criteria for PD patients (12). According to the main eligibility criteria of the PPMI protocol, all PD subjects were Hoehn and Yahr stage I or II at baseline. Patients must have had at least two of the following: resting tremor, bradykinesia, rigidity (must have either resting tremor or bradykinesia) or either asymmetric resting tremor or asymmetric bradykinesia. All subjects had Geriatric Depression Scale scores within normal ranges. In addition, exclusion criteria included subjects who received any of the following drugs that may interfere with DA transporter SPECT imaging: neuroleptics, metoclopramide, alpha methyldopa, methylphenidate, reserpine, or amphetamine derivative, within 6 months of screening. Current treatment with anticoagulants (e.g., coumadin, heparin) that might preclude safe completion of the lumbar puncture was another exclusion criteria. Additional demographic data about the subjects can be found in Table 1.

**Table 1:** **Demographic information**.

	Parkinson’s disease subjects	Controls	*p*-value
Age, SD (years)	62, 9	60, 12	0.59
Gender (% male)	53.3	58.8	0.809
Caucasian (%)	100	94.1	0.3605
Right-handed (%)	96.7	100	0.0806

Initially, 265 potential subjects were screened and rated for the clarity of the SN and nigrosome-1. A strict quality-control process was imposed during subject selection to improve the quality of the manual segmentation analysis, the next step of the analysis. The quality-control process considered both signal-to-noise ratios and dynamic range in contrast and was done without knowledge of the cohort to which each subject belonged. In addition, the selection criteria were as follows: (1) axial scan acquisition, (2) 3-T field strength, and (3) the visualization of the nigrosome-1 as a region of signal hyperintensity in the posterior third of the SN surrounded by low signal intensity anterior and laterally (pars compacta SN) and medially by low signal from the medial lemniscus. Because this study involved manual segmentation, to avoid inaccuracy, two separate, blinded raters determined that the scans of 50 subjects among the 265 had a sufficiently visible nigrosome-1 to proceed with the analysis. The two raters were blinded as to whether the subjects were of the PD or control cohort. Therefore, only 50 subjects were deemed appropriate for the subsequent segmentation analyses based upon the strict quality-control process and selection criteria imposed upon the images, as a measure to improve the quality of the future analyses and reduce the likelihood of inaccuracy during manual segmentation. Three subjects scanned on the Discovery MR750 machine model were then removed from further analyses to reduce possible heterogeneity of scanner hardware.

### Image Acquisition and Scanning

Subjects were scanned at 3 T using T2-weighted, axial acquisition sequences with a scan time of 5 min and 8 s. MRI data was acquired on either a Siemens TrioTim system or GE Medical Systems SignaHDxt system. The resolution ranged from 0.49 mm × 0.49 mm × 2.0 mm to 0.94 mm × 0.94 mm × 3.0 mm, TR (repetition time) ranged from 3000 to 4500 ms, TE (echo time) ranged from 11 to 105.98 ms, flip angle was either 90° or 150°, and matrix size ranged from 204 × 256 × 48 voxels to 512 × 512 × 84 voxels.

### Behavioral Measures

Behavioral measures were compiled using the PPMI database. Movement Disorder Society-Unified Parkinson’s Disease Rating Scale (MDS-UPDRS) subscores were calculated and compiled for each subject. Part I (non-motor) scores measured non-motor experiences of daily living consisting of Subscore IA (nBehav), which focused on complex behaviors, and IB (nExp), which focused on the patient’s experiences regarding non-motor experiences of daily living; Part II (mExp) measured motor experiences of daily living; and Part III (mSign) measured motor signs of PD (13). The tests that composed Subscores IA and IB can be found in Table 2.

**Table 2:** **The six different tests that composed Subscore IA (nBehav) and the seven different tests that composed Subscore IB (nExp)**.

Part IA: complex behaviors (nBehav)	Part IB: patient experiences (nExp)
Cognitive impairment	Sleep problems
Hallucinations and psychosis	Daytime sleepiness
Depressed mood	Pain and other sensations
Anxious mood	Urinary problems
Apathy	Constipation problems
Features of dopamine dysregulation syndrome	Light headedness on standing
–	Fatigue

**Figure 1:**
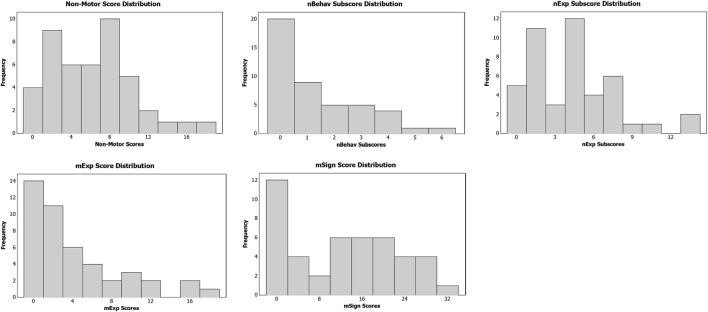
**Distribution of clinical MDS-UPDRS data**.

The results of the MDS-UPDRS clinical measures demonstrated a median score of 7 and range of 0–19 for non-motor behaviors and experiences, a median score of 2 and range of 0–17 for motor experiences, and a median score of 13 and range of 0–36 for motor signs. The distribution of the clinical data can be found in histogram format in Figure 1.

### Image Processing

Three approaches were used to locate the SN region and nigrosome-1 region: manual segmentation, automated segmentation, and area voxel-based morphometry. Details of the three approaches are as follows.

#### Manual Segmentation

**Figure 2:**
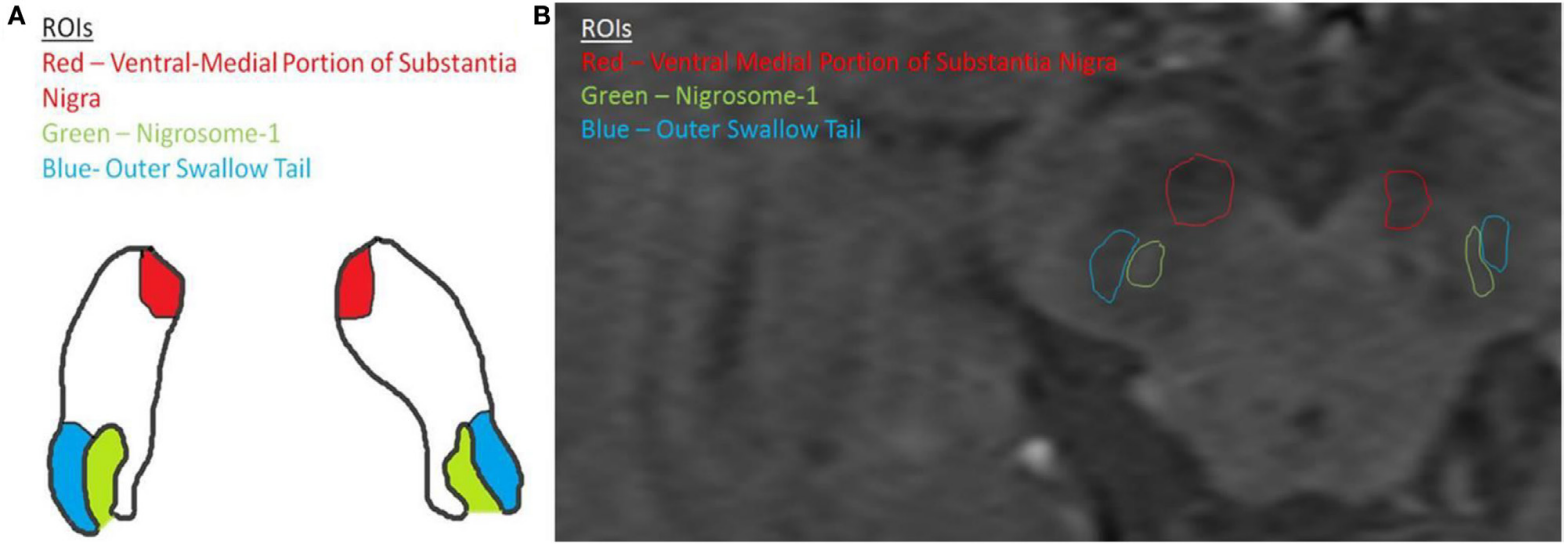
**(A)** Schematic diagram of ROIs and **(B)** ROIs as seen on patient MRI scan.

The manual segmentation component involved the manual segmentation of regions of interest (ROIs) following a standardized protocol. The SN and nigrosome-1 were manually segmented by two separate experts who had followed the protocol below when conducting the segmentation. The most inferior axial slice where the red nucleus was still visible was used for the segmentation of the SN and nigrosome-1, and this was done uniformly for all subjects. The SN was located in the mesencephalon, posterior (dorsal) to the crus cerebri, anterior (ventral) to the midbrain tegmentum, and inferolaterally to the red nucleus (14). As previously described, it lies at an oblique angle with its superior boundary most lateral. It is typically divided into two functionally and anatomically distinct parts: the inferior (caudal) and posterior (dorsal) SN pars compacta (SNc), which has melanin-containing neurons, and the superior (rostral) and anterior (ventral) SNr (15). Based on previous MR studies (15), the SN pars reticulata was identified as the region of signal hypointensity on T2 in the midbrain. The nigrosome-1 was identified as the signal hyperintensity in the posterior third of the SN and was surrounded by low signal intensity anteriorly and laterally by relatively low signal from the cerebral peduncles and medially by low signal from the red nucleus and SN pars reticulata (15) (Figure 2).

#### Automated Segmentation of Substantia Nigra

All normal controls’ T2-weighted images were coregistered to build a template structural image, which then served as the common space for automated segmentation. The spatial correspondences, established between subject images and the template image with non-linear registration, were used to propagate manual segmentation results. Manual segmentation of three subject images selected at random out of the total sample was mapped to the template space and averaged. This averaged segmentation in the template space was propagated to all subject images, including the three original input subject images. Segmentations of the three subjects’ images were therefore regenerated with the automated segmentation protocol.

Non-linear registration was performed with Advanced Normalization Tools (ANTs) registration (16). The ANTs registration tool first performs linear alignment of images to correct orientation mismatch, then performs non-linear deformation to register images. In our experience, ANTs is very robust in handling orientation mismatch, as long as the images are not turned beyond 90 degrees.

**Figure 3:**
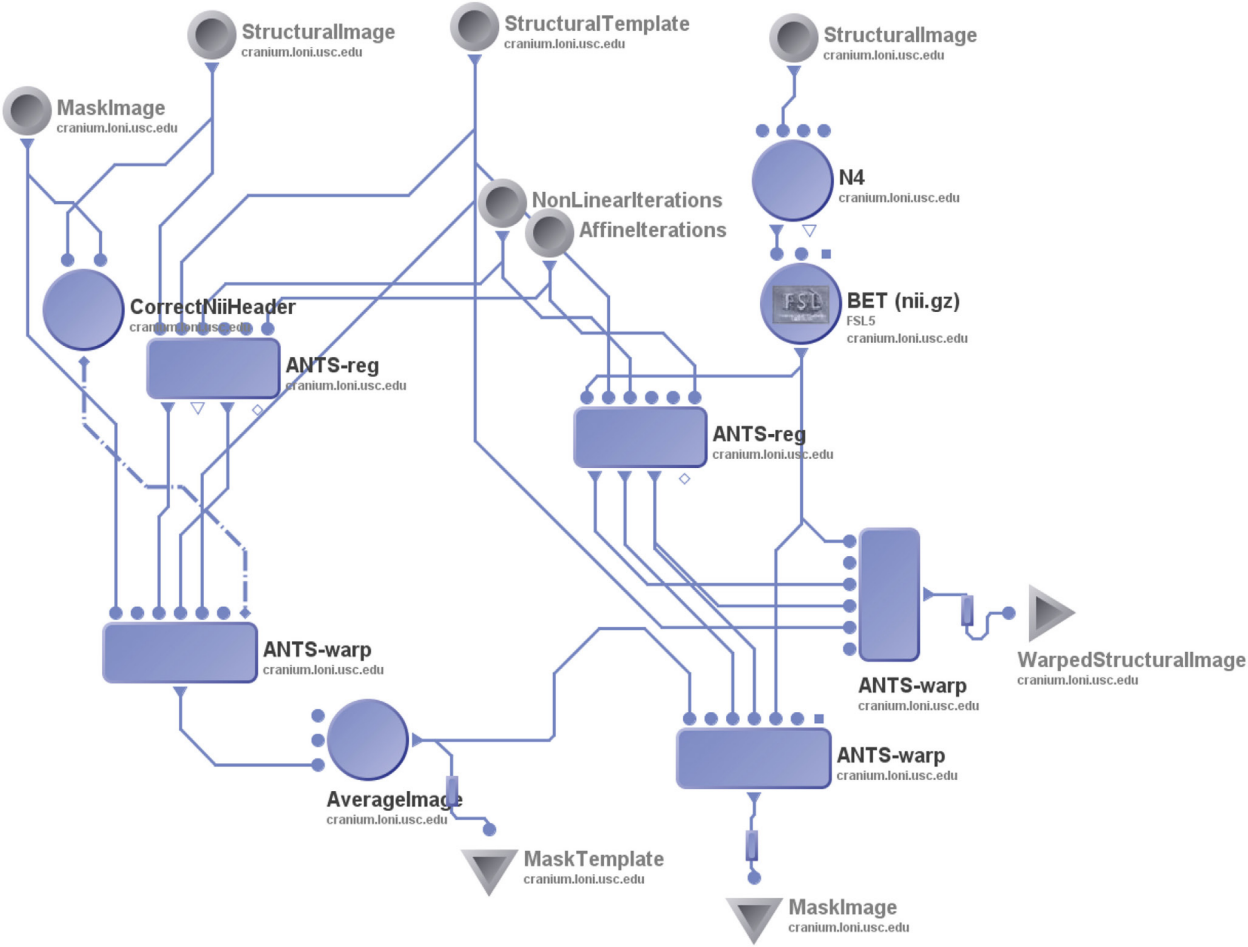
**Pipeline workflow illustrating the automated segmentation process**.

As a preprocessing step of spatial normalization, all T2-weighted images were skull-stripped with the FSL5 Brain Extraction Tool (BET) version 5.0.2 (17). This automated segmentation pipeline, as illustrated in Figure 3, output segmentations of the SN (excluding the nigrosome-1) of the remaining T2-weighted images (18, 19). The nigrosome-1 was then manually determined based on the gap left within the SN segmentation.

The pipeline workflow includes two complementary components, divided by the red dashed line in Figure 3. The left side of the pipeline constructs the SN in the atlas image. Module R1 uses subjects’ structural images from Input B and non-linearly registers them to the structural atlas specified by Input A. Module W1 then takes the non-linear transformations output by Module R1 and applies them to warp the manually segmented SN binary masks (Input A) into the atlas space. These spatially normalized masks are averaged to produce Output E, which is the averaged SN in the atlas space. Input A and B are both skull-stripped images. The right side of the pipeline, as illustrated in Figure 3, propagates the SN region from the atlas space to subjects’ native spaces. Input D includes the structural images of the 47 subjects. After correction for field inhomogeneity and skull stripping, they are registered to the atlas space by Module R2. Module R2 outputs a two-way transformation: the forward warp from subject spaces to the atlas space and the inverse warp from the atlas space to subject spaces. Module W2 uses the inverse transformations and applies them to propagate the SN segmentation from the atlas to each of the subjects’ spaces as Output F, which yields the results of the automatic segmentation. Module W3 takes the forward transformations and applies them to warp subject images to the atlas space for registration quality control. Please note that Pipe G feeds the segmentation in the atlas into Module W2, connecting the left part and the right part of the workflow. The atlas input C, shared by both the left and the right parts, is constructed from structural images of all normal control subjects, with ANTs, in a way similar to that for the construction of the Montreal Neurological Institute (MNI) atlas.

In Modules R1 and R2, the number of non-linear registration iterations was set to 50 × 50 × 50, while linear iterations were set to 1000 × 1000 × 1000. The energy function used was “mutual information,” and the transformation model used was “SyN.”

#### Area Voxel-Based Morphometric Approach

The area voxel-based morphometric (aVBM) approach utilized an automated segmentation process as well. The same template structural image created for the automated segmentation process was used for the aVBM approach. This aVBM approach used circular ROIs as opposed to a segmentation approach that yielded ROIs based on the anatomy of the SN and nigrosome-1. ROIs were drawn for four subjects selected randomly from the total sample in three regions of the SN: (1) nigrosome-1, (2) ventromedial region of the SN, and (3) outer “swallow tail” of the SN, a region dorsolaterally adjacent to the nigrosome-1. The spatial correspondences established between subject images and the template image with non-linear image registration were used to segment these 3 ROIs on all 47 subjects, as was done in the automated segmentation protocol using the same pipeline (Figure 3). The voxel intensity ratios were then calculated from these ROIs.

### Voxel Intensity Analyses

Two voxel intensity ratios were calculated (IR1 and IR2) using the most inferior axial slice where the red nucleus is still visible. To reduce intensity variability of a single voxel, five voxels were randomly sampled from each ROI. The region spanned by the five voxels covers 60–100% of the ROI. Therefore, the mean of the five voxels adequately represented the intensity of the ROI. Let rNigro1 and lNigro1 denote the set of voxel intensities sampled from the nigrosome-1 on the right and left sides, respectively, rVentral and lVentral denote those from the ventral–medial portion of the SN on the right and left sides, respectively, and rSwallow and lSwallow denote those from the outer swallow tail on the right side and left side, respectively (Figure 2). IR1 and IR2 were calculated as follows:

$$\text{IR}1=\frac{\frac{\text{mean}(\text{rNigro}1)}{\text{mean}(\text{rVentral})}+\frac{\text{mean}(\text{lNigro}1)}{\text{mean}(\text{lVentral})}}{2}\;\text{and}\;\text{IR}2=\frac{\frac{\text{mean}(\text{rNigro}1)}{\text{mean}(\text{rSwallow})}+\frac{\text{mean}(\text{lNigro}1)}{\text{mean}(\text{lSwallow})}}{2}$$

Because IR1 and IR2 are ratios of image intensities, the scanners used for image acquisition should have a limited impact on these indices.

### Statistical Analysis

#### Multivariate Multiple Regression Analysis

We employed descriptive statistics, multivariate analysis of variance, and linear regression to examine the relationships between predictive imaging biomarkers and clinical PD measures (20, 21). In our linear regression models, the explanatory variables *X* include the intensity ratios, IR1 and IR2, derived from the nigrosome-1 in the SN. The responding variables (*Y*) include four MDS-UPDRS measures: nBehav, nExp, mExp, and mSign, as defined below:

- MDS-UPDRS:
  ◦ Part I: non-motor experiences of daily living;
    ▪ Subscore IA: complex behaviors (nBehav);
    ▪ Subscore IB: non-motor experiences of daily living – patient experiences (nExp).
  ◦ Part II: motor experiences of daily living (mExp);
  ◦ Part III: motor signs of PD (mSign).

We used power analysis (21, 22) to estimate the statistical power of our multivariate linear model. Using default false-positive rate of *a* = 0.05, the available group sample sizes (controls: 17 and patients: 30), effect-size of 0.70, and assuming relative independence of the predictors (unitary variance inflation factors) and equal variability in each cohort, we estimated that the power to detect a grouping effect is 0.95.

## Results

### Slice Thickness

**Figure 4:**
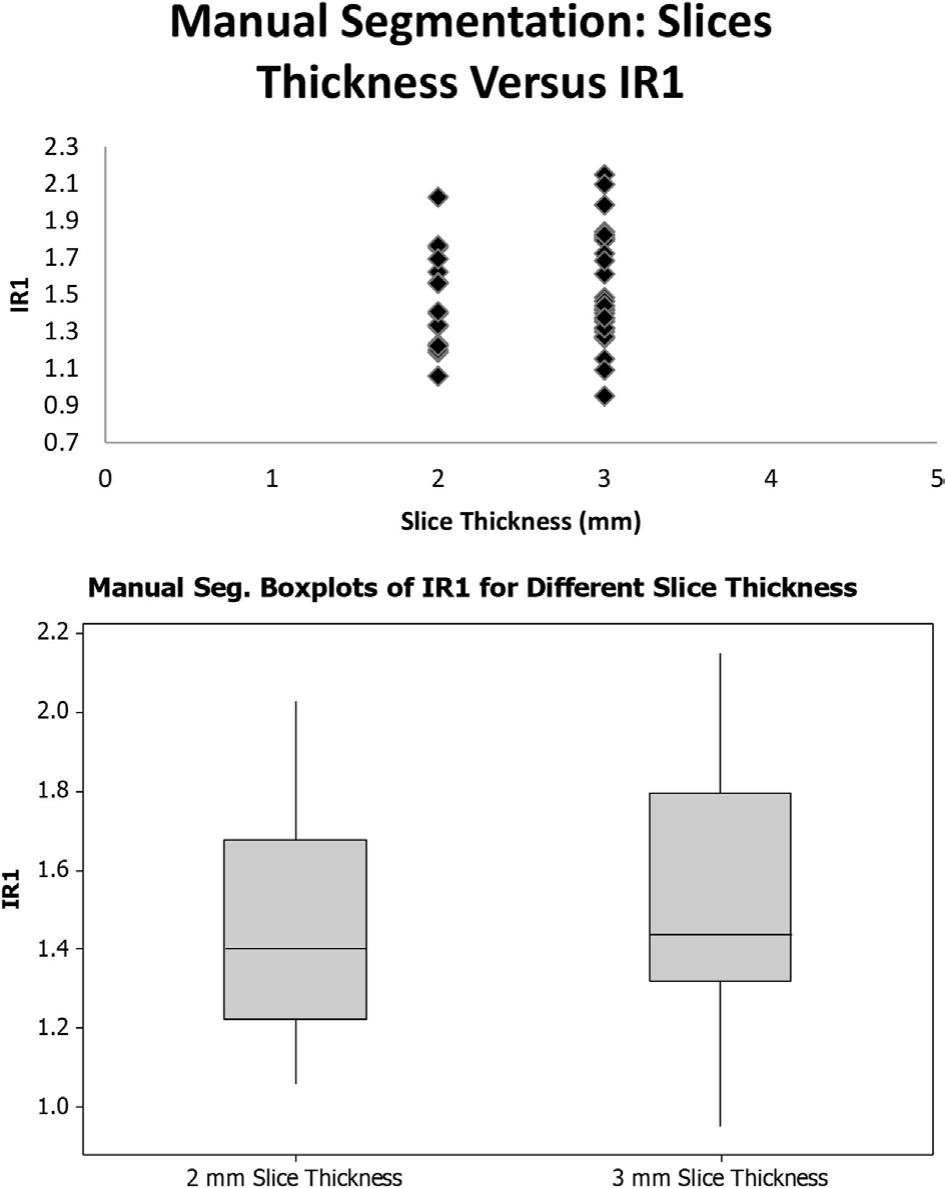
**Slice thickness and voxel intensity ratios (IR1)**.

**Figure 5:**
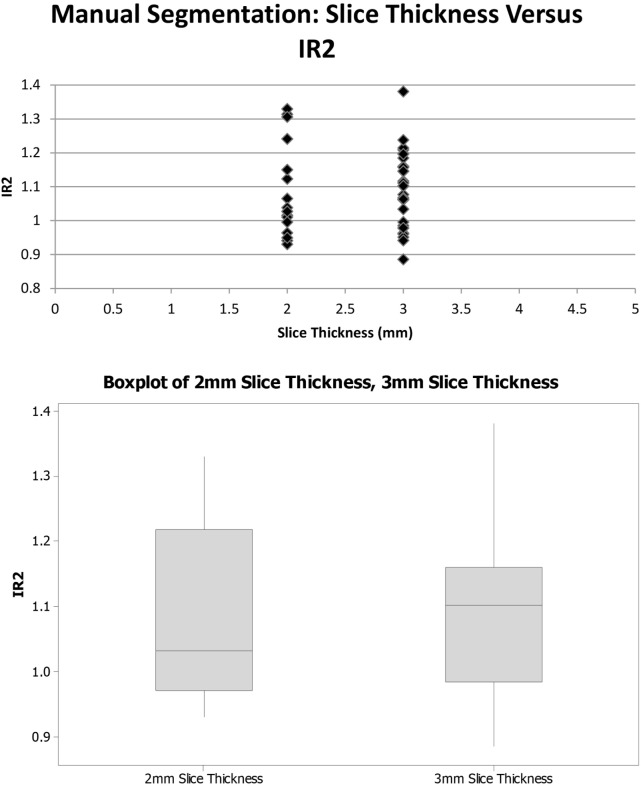
**Slice thickness and voxel intensity ratios (IR2)**.

To address the concern that the different scanner types and slice thicknesses may potentially affect the results of the voxel intensity ratios, scatterplots were created to demonstrate that the differences in scan acquisition and slice thickness yielded a similar distribution of data (Figures 4 and 5).

### Linear Modeling

#### Manual Segmentation

The results of fitting the multivariate multiple regression model for the manual segmentation analyses indicate that the predictor vector of intensity ratios, *X* = {IR1, IR2}, is border-line significant in explaining the observed response (clinical PD measurements) *Y, p* = 0.04956. Fitting a linear regression model on the individual responses (*Y* ~ IR1 + IR2) showed that linear regression tests consistently identified the IR2 as predictive of nBehav (*p* = 0.0377) and nExp (*p* = 0.03856).

#### Automated Segmentation

For automated segmentation, multivariate multiple linear regression analysis to predict the response vector *Y* = {nBehav, nExp, mExp, mSign} from the imaging biomarkers *X* = {IR1, IR2} did not provide significant evidence of association (MANOVA Wilks test, *p* = 0.09564). However, linear regression tests consistently identified the IR2 as predictive of nBehav (*p* = 0.01), nExp (*p* = 0.00336), mExp (*p* = 0.02125), and mSign (*p* = 0.008139).

#### Area Voxel-Based Morphometric Analysis

Multivariate multiple linear regression analysis to predict the response vector *Y* = {nBehav, nExp, mExp, mSign} from the imaging biomarkers, *X* = {IR1, IR2} provided significant results for nBehav (*p* = 0.008991), mExp (*p* = 0.04869), and mSign (*p* = 0.01489). For univariate simple linear regression analysis, IR2 yielded significant results for nBehav (*p* = 0.003102), mExp (*p* = 0.0172), and mSign (*p* = 0.00393).

## Discussion

Previous research on the nigrosome-1 has suggested that visualization of this high signal intensity wedge may have the potential to serve as a simple, sensitive, and specific diagnostic tool for PD, using either 7 or 3 T T2*-weighted SWI sequences (5, 6, 23). These investigations used blinded radiologists or neurologists to determine the diagnostic accuracy of this type of visual radiologic assessment, predicting whether subjects were diagnosed with PD or were healthy controls. Nevertheless, T2-weighted images are often obtained in routine clinical MRI protocols and are thereby more readily accessible. In contrast to these studies, we therefore aimed to determine whether the nigrosome-1 could serve as a more readily accessible, potential biomarker for PD using T2-weighted images, in that changes in the nigrosome-1 correspond with signs of PD progression as assessed by the MDS-UPDRS.

The ability to visualize the nigrosome-1 on MRI has been associated with the T2*-weighted SWI sequence’s sensitivity for iron (24). T2-weighted imaging, while not as sensitive to iron as SWI sequences, is also sensitive to local magnetic field inhomogeneities and modified in the presence of iron, with previous studies suggesting its potential to serve as a non-invasive estimate of iron content in the brain (7–9). Whereas the majority of studies investigating the nigrosome-1 and its degeneration in PD have focused on its direct visualization with 7 or 3 T T2*-weighted SWI sequences, we demonstrated positive findings even when using T2-weighted images. Because sensitivity of T2 is less than SWI, the positive findings here were more conservatively derived than those of SWI. Recent research has focused on improved imaging of the SN, including the use of new MRI techniques, such as “neuromelanin-sensitive MRI” (NM-MRI) and quantitative susceptibility mapping (QSM), which have been found to provide notable contrast between the SN and surrounding brain tissues with potential applications as biomarker of PD (25, 26). While these innovative neuroimaging techniques continue to improve the resolution of SN imaging, standard structural T2- and T1-weighted MRIs are often normally acquired during early PD. T2-weighted imaging may therefore be more prevalent and readily available than T2*-weighted SWI sequences (27). The results of this investigation suggest its potential applicability to the development of a more accessible neuroimaging diagnostic tool for PD patients, as T2-weighted MRI images are routinely collected as a part of standard imaging protocol in comparison to SWI or T2* images.

In this study, we selected subjects with neuroimaging data providing sufficient detail for automated extraction of the nigrosome-1. This protocol avoids biases and inconsistencies of manual segmentation that may otherwise influence the first component of this study. To enable the comparison of the automatic segmentation results and their manual segmentation counterparts, we used a paired design with the same subjects processed *via* both approaches. Previous studies have used automatic segmentation techniques in morphometric studies relating to neurological diseases (28, 29), while other studies employed a combination of manual and automated segmentation techniques, similar to this investigation (30, 31). The results of the automatic segmentation approach in this analysis demonstrated a stronger association between the voxel intensity ratios and clinical measures of PD than the manual segmentation approach. Computer-based automatic segmentation, more quantitative than human manual segmentation, may be able to detect the nigrosome-1 even when it is difficult by visual inspection. Therefore, the addition of an automated segmentation approach to the manual segmentation approach alone in this analysis likely reduced inter- and intra-rater segmentation quality incoherence. We will further compare automatic segmentation and manual segmentation of nigrosome-1 in future studies.

Further, by calculating voxel intensity ratios instead of using absolute voxel intensity values, we accounted for changes in iron levels throughout the basal ganglia that may occur with aging (32). Nevertheless, while our hypothesis is originally based upon previous literature that suggests that changes in the nigrosome-1 in PD patients may be reflective of changes in iron levels in the basal ganglia in PD, the manner by which T2-weighted MRI changes in the nigrosome-1 relate to clinical measures of PD remains unclear. While T2-weighted MRI’s susceptibility to iron inhomogeneities may play a role, other factors may also contribute, which merit further elucidation by future studies.

The results of our statistical analyses revealed that IR2, derived from the nigrosome-1 and the area immediately dorsolateral to it, seemed to be more impactful and better correlated with MDS-UPDRS scores than IR1, derived from the nigrosome-1 and a ventromedial area of the SN. Similarly, the results of the linear regression analysis for the automated segmentation component of the analysis demonstrated that IR2 was a significant predictor of the MDS-UPDRS scores but IR1 was not. This finding may be related to the pattern of dopaminergic loss in the SN in PD patients, as dopaminergic neuronal loss is most pronounced in the caudal and ventrolateral tier, with the dorsal tier being the most preserved (3, 33, 34). Dopaminergic neuron loss is highest in the nigrosomes, with maximal loss (98%) in nigrosome-1 (3), and then the loss progresses to other nigrosomes and the remaining matrix along rostral, medial, and dorsal axes. In this case, a ratio derived from the nigrosome-1, experiencing the highest levels of dopaminergic loss, and the dorsolateral SN, experiencing minimal loss, has the greatest likelihood of corresponding with PD progression as captured by MDS-UPDRS scores. In contrast, a ratio derived from the nigrosome-1 and a ventromedial area of SN, which may also be experiencing certain levels of dopaminergic loss, would not correspond as well.

One might expect the imaging biomarkers to show the strongest correlation with Part III scores, which is the motor examination of the MDS-UPDRS conducted by a physician. However, partial correlations between IR2 and all score components of the MDS-UPDRS either in the manual segmentation analysis or in the automated segmentation analysis were significant. This suggests that voxel intensity ratios have the potential to not only relate to motor signs of PD, as assessed by a third-party examiner that is not the caregiver or patient, but also relate to self-reported patient questionnaires.

While changes in PD patients may be associated with changes in iron content in the nigrosome, other possible mechanisms involved include neuronal cell loss, changes in neuromelanin, a change in iron oxidation state, or a combination of these effects (5). While this study is based upon T2 sequence sensitivity for iron, it does not elucidate the exact mechanism of the changes in the nigrosome-1 that correspond with PD progression. Additional research is needed to further elucidate these mechanisms.

The results of this investigation support previous research, suggesting that the nigrosome-1 as a region of signal hyperintensity may relate to the symptoms seen in PD. More specifically, this study suggests that there is an association between T2-weighted MRI changes in the nigrosome-1 and clinical measures of PD as assessed by the MDS-UPDRS. There has also been recent interest in determining whether the nigrosome-1 is capable of differentiating different neurodegenerative Parkinsonism presentations, including PD, multiple system atrophy (MSA), and progressive supranuclear palsy (PSP) (23). Additional studies can investigate whether changes in the nigrosome-1 correlate with clinical manifestations of MSA or PSP in addition to PD.

We demonstrate support for the *a priori* hypothesis that voxel intensity ratios derived from T2-weighted imaging may serve as predictors of MDS-UPDRS scores based on the results of our linear regression analyses. The voxel intensity ratio derived from the dorsolateral SN and nigrosome-1 was consistently predictive of non-motor complex behaviors in all three analyses and predictive of non-motor experiences of daily living, motor experiences of daily living, and motor signs of PD in two of the three analyses. T2-weighted images are often obtained more readily in routine clinical MRI protocols than other sequences. Our results suggest that T2 changes in the nigrosome-1 may be considered when developing a more accessible clinical diagnostic tool for patients with suspected PD in the future.

## Author Contributions

KF was involved in the design and conceptualization of the study, analysis or interpretation of the data, and drafting and revising the manuscript for intellectual content. RN and Dr. JL were involved in the analysis of the data and revising the manuscript for intellectual content. Dr. ID was involved in the statistical design and analysis of the data, as well as drafting and editing the manuscript. Dr. AT was involved in the design and conceptualization of the study, analysis and interpretation of the data, and revising the manuscript for intellectual content.

## Conflict of Interest Statement

The authors declare that the research was conducted in the absence of any commercial or financial relationships that could be construed as a potential conflict of interest.
